# Association of Dietary Fiber, Composite Dietary Antioxidant Index and Risk of Death in Tumor Survivors: National Health and Nutrition Examination Survey 2001–2018

**DOI:** 10.3390/nu15132968

**Published:** 2023-06-29

**Authors:** Zongbiao Tan, Yang Meng, Lu Li, Yanrui Wu, Chuan Liu, Weiguo Dong, Changzheng Chen

**Affiliations:** 1 Department of Gastroenterology, Renmin Hospital of Wuhan University, 238 Jiefang Road, Wuhan 430060, China; 2 Department of Ophthalmology, Renmin Hospital of Wuhan University, 238 Jiefang Road, Wuhan 430060, China

**Keywords:** dietary fiber, composite dietary antioxidant index, cancer survivors, mortality

## Abstract

Background: Dietary fiber is a functional substance with strong antioxidant activity that plays an important role in human health. Dietary fiber has been shown to reduce the risks of many types of cancers, but whether it can reduce the risk of death in cancer survivors remains undetermined. Methods: This study included the dietary data of cancer survivors who participated in the National Health and Nutrition Examination Surveys from 2001 to 2018. Firstly, the relationship between fiber intake and composite dietary antioxidant index (CDAI) was explored by weighted multiple regression and smooth curve. Subsequently, multivariable Cox proportional hazards regression models were used to explore the effects of dietary fiber intake and CDAI level on the risks of all-cause, tumor, and cardiovascular death among cancer survivors. Results: A total of 2077 participants were included in the study, representing approximately 11,854,509 cancer survivors in the United States. The dietary fiber intake of tumor survivors had a nonlinear positive relationship with CDAI levels (β = 0.24, 95% CI: 0.08–0.40, *p* = 0.004). Multivariable Cox proportional hazards regression models showed that high dietary fiber intake and CDAI levels were associated with reduced risks of all-cause and tumor death in tumor survivors, but were not associated with the risk of cardiovascular death. Conclusion: An increased dietary fiber intake can enhance the body’s antioxidant capacity. A higher dietary fiber intake and CDAI level may reduce the risk of all-cause and tumor death in tumor survivors.

## 1. Introduction

Malignant tumors are one of the major public health problems all over the world. According to the World Health Organization (WHO), there were 19.3 million new cancer cases and nearly 10 million cancer deaths worldwide in 2020 [1]. In most countries, tumors are already the second leading cause of death after cardiovascular diseases and are expected to be the main obstacle to life expectancy growth in the 21st century [2].

Various dietary components (including energy and nutrients) can affect the inflammatory process in the human body, leading researchers to focus on the relationship between diet and tumors [3,4]. Previous studies have focused on the effect of diet on tumor incidence. For example, processed meat and red meat can increase the risk of gastric cancer, and a high-fat diet may affect bile acid metabolism, leading to a higher risk of colon cancer [5,6]. However, few studies have looked at the effects of diet on patients who have already suffered from tumors. In fact, chronic inflammatory status can also be improved through diet, the most representative of which is the Mediterranean diet, which is dominated by a plant-based diet [7,8]. Recently, a newly published study by the National Cancer Institute found that dietary fiber can improve the treatment effect of melanoma treated with immune checkpoint inhibitors [9]. As an important part of the Mediterranean diet, dietary fiber is listed as the “seventh largest nutrient” after sugar, protein, fat, water, minerals, and vitamins. With the deepening of the understanding of dietary fiber, researchers have found that dietary fiber has a variety of health benefits, such as preventing the occurrence of colorectal cancer and reducing the risk of death in the general population [10,11,12]. However, there are no studies evaluating the contribution of dietary fiber intake to the body’s antioxidant capacity. The Complex Dietary Antioxidant Index (CDAI) is a measurement of dietary antioxidant capacity based on the contributions of a variety of antioxidant components (selenium, carotenoid, zinc, vitamin A, vitamin C, and vitamin E) [13,14].

In this study, we aim to explore the relationship between dietary fiber intake and CDAI and to evaluate the effects of dietary fiber intake and CDAI on the prognosis of tumor survivors, hoping to provide new insights into nutrition and supportive care for cancer patients.

## 2. Materials and Methods

### 2.1. Data Sources

National Health and Nutrition Examination Surveys (NHANES) is a cross-sectional sampling survey conducted by the National Center for Health Statistics (NCHS). It collects dietary and medical conditions about the general population, and is used to monitor the health and nutritional status of the national population [15]. A total of nine cycles (2001–2018 years) of NHANES data were obtained in this study.

### 2.2. Study Population

From 2001 to 2018, a total of 4780 people completed the question “Ever told you had cancer or malignancy?” in the Medical Conditions section of the questionnaire, and replied with “Yes”. The selection criteria for tumor survivors are as follows: (1) exclude age < 40 years old; (2) exclude incomplete age information for the first diagnosis of tumor; (3) exclude participants with two or more types of tumors; (4) exclude participants with incomplete information on diet, smoking, alcohol use, and chronic diseases.

### 2.3. Diet Assessment

Dietary data are rough intakes of energy, nutrients, and other components obtained by counting the types and amounts of foods and drinks (including water) consumed by participants in the 24 h prior to the interview. And the interview was carried out twice for each participant. In this study, data on energy (kcal), protein (mg), carbohydrate (mg), dietary fiber (mg), total fat(mg), cholesterol (mg), and six antioxidants (selenium, carotenoid, zinc, vitamin A, vitamin C, and vitamin E) were extracted. The average of the two diets was used as the participants’ daily intake. CDAI was calculated based on the formula proposed by Wright et al. [13]. To estimate CDAI, we first standardized the dietary intake of the six antioxidants (vitamins A, C, and E, selenium, carotenoid, and zinc) by subtracting the global average and dividing by the global standard deviation. Then, we calculated the CDAI, summing up the standardized intakes of these antioxidants with equal weight as described below:

(1) $$\mathrm{CDAI}=\overset{6}{\underset{i=1}{\sum }}\frac{\mathrm{Xi}-\mu i}{\mathrm{Si}}$$

Xi represents the daily intake of antioxidant i (i.e., vitamins A, C, and E, selenium, carotenoid, and zinc), μi represents the mean Xi of the whole cohort for antioxidant I, and Si represents the standard deviation for μi.

### 2.4. Ascertainment of Covariates

To exclude the interference of other confounding factors on death outcomes, we adjusted for age, sex, race, education, body mass index (BMI), bad lifestyle behaviors (smoking and alcohol consumption), and chronic diseases (hypertension, diabetes, coronary heart disease (CHD), and stroke). This information came from questionnaires and laboratory measurements, as described below: race (including White, Black, Mexican Americans, and others); education level (including less than high school education, high school education, and some college or associate of arts degree); BMI (<18.5, 18.5–25, ≥25). Bad lifestyle behaviors included smoking (never, former, and now) [16], and the classification of alcohol use is as follows: never, former, mild, moderate, and heavy. Chronic diseases included diabetes, hypertension, CHD, and stroke, with the first being categorized as diabetes mellitus (DM), compromised fasting glycemia, and impaired glucose tolerance, while the latter three were based on the patient’s self-report [17].

### 2.5. Ascertainment of Outcomes

NCHS provided NHANES-associated mortality documentation as of 31 December 2019. The causes of death of the participants were classified according to the 10th International Classification of Diseases, including death from cardiovascular diseases (I00–I09, I11, I13, I20–I51), cancers (C00–C97), and other causes. Survivor’s survival time (months) is defined as the number of years from the age of first cancer diagnosis to death multiplied by 12.

### 2.6. Statistical Analysis

To ensure that the data we collected were representative of overall cancer survivors in the United States, we followed the NCHS guidelines for opinion selection weights. Firstly, we described the baseline characteristics of cancer survivors based on their final survival status. For continuous variables (age, carbohydrate, energy, protein, total fat, dietary fiber, and CDAI), means and standard errors were presented, while counts and proportions (after weighting) were presented for categorical variables (sex, race, education level, smoke, alcohol user, diabetes, hypertension, stroke, and CHD). Student’s *t*-tests were used to compare continuous variables and chi-square tests were used to compare categorical variables. Subsequently, we used a weighted multiple linear regression model to evaluate the relationship between dietary fiber intake and CDAI scores, and used smooth curves to fit potential nonlinear associations between them. Multivariate Cox risk regression models were used to estimate the hazard ratio (HR) and 95% confidence interval (CI) of dietary fiber intake and CDAI score for risk of all-cause, tumor, and cardiovascular death. In addition, dietary fiber intake was divided into <25 g/day, 25–29 g/day, and >29 g/day according to the recommended intake of dietary fiber [10], and CDAI was classified using the quartile method. Then, the relationship between different dietary fiber intake levels and CDAI levels and risk of death was evaluated again by adjusting confounding factors. All statistical analyses were performed using R (version 4.2.3). The smooth curve was finished using EmpowerStats (www.empowerstats.com). (accessed on 28 March 2023) *p* < 0.05 was considered statistically significant.

## 3. Results

**Figure 1:**
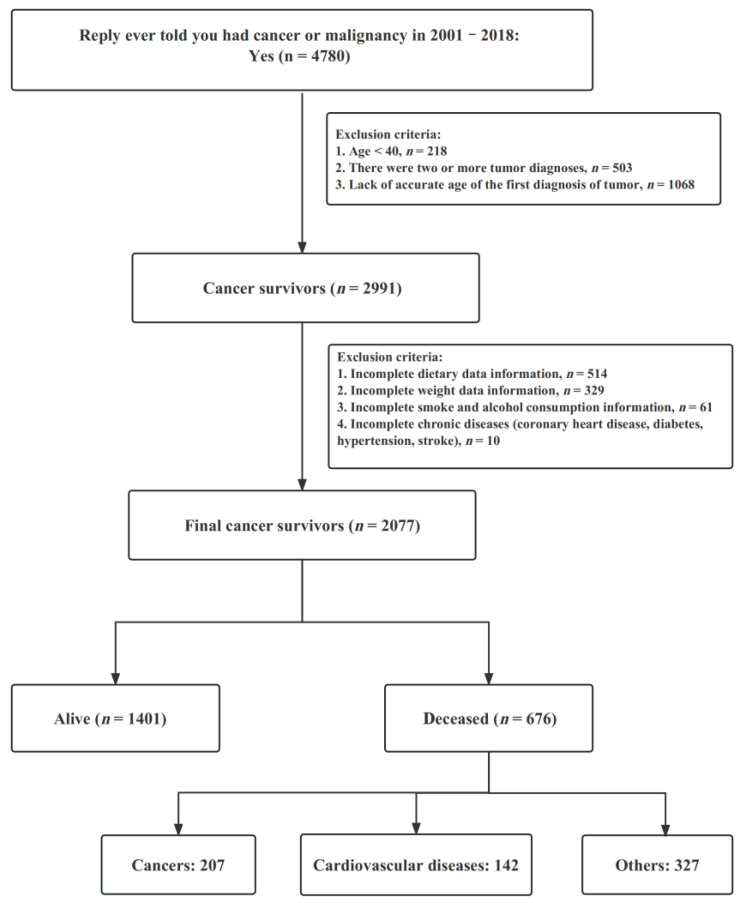
Flow chart of sample selection from the NHANES 2001–2018.

Using the above exclusion criteria, 2077 cancer survivors were finally included at baseline (Figure 1). During the follow-up period (median time 82 months), 676 deaths occurred, including 207 from neoplastic causes and 142 from cardiovascular causes, followed by chronic respiratory diseases, cerebrovascular events, and others.

### 3.1. Population Characteristics

The 2077 cancer survivors included in this study represent an estimated 11,854,509 cancer survivors in the United States. Table 1 shows the characteristics of baseline samples classified by the survival status of cancer survivors. In this study, male individuals occupied a significantly higher proportion of the deceased individuals, compared with female ones (*p* = 0.002). Moreover, patients who died were older and had lower intakes of energy, protein, total fat, and especially dietary fiber compared with those who did not (all *p* < 0.05). In addition, CDAI levels, which represent the body’s antioxidant capacity, were much smaller in deaths than in survivors (−0.12 and 0.60, respectively; *p* < 0.001). Common factors such as smoking, alcohol consumption, hypertension, stroke, and CHD are high risk factors for death (all *p* < 0.05). However, for BMI, the difference between the two groups approached statistical significance (*p* = 0.05). There was no significant difference in carbohydrate, total sugars, and total cholesterol intake.

### 3.2. Relationship between Dietary Fiber Intake and CDAI

**Figure 2:**
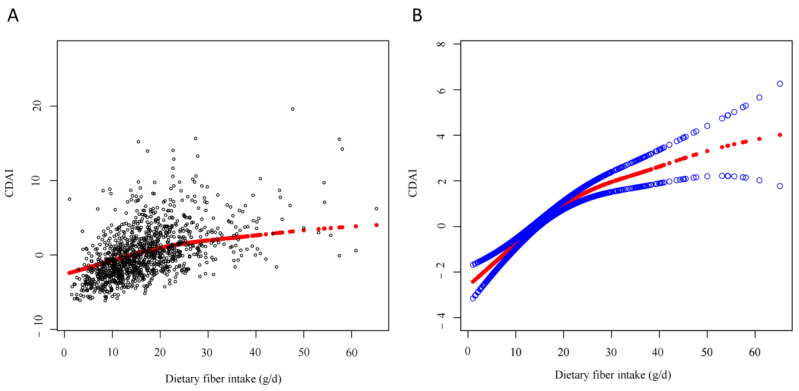
Smooth curve fitting of daily dietary fiber intake to CDAI. (**A**) Each black dot represents a sample. (**B**) The red line indicates a smooth curve fitting between daily dietary fiber intake and CDAI. The blue line represents the 95% confidence interval for the fit. Adjusted for age, sex, race, education level, smoke, alcohol intake, carbohydrate, energy, cholesterol, protein, and total fat. CDAI = composite dietary antioxidant index.

We constructed three weighted multiple linear regression models and consistently observed a positive correlation between dietary fiber intake and CDAI levels (Model 1: β = 0.28, 95% CI: 0.17–0.38, *p* < 0.001; Model 2: β = 0.27, 95% CI: 0.16–0.38, *p* < 0.001; Model 3: β = 0.24, 95% CI: 0.08–0.40, *p* = 0.004; Table 2). Subsequently, we converted dietary fiber intake from a continuous variable to a categorical variable and into three groups (<25 g/day, 25–29 g/day, and >29 g/day). We also fully adjusted for age, sex, race, education level, smoking, alcohol intake, carbohydrate, energy, cholesterol, protein, and total fat. Finally, we found that, compared to patients with dietary fiber intake <25 g/day, CDAI increased 1.2 (95% CI: 0.37–2.02, *p* = 0.004) and 1.99 (95% CI: 0.75–3.24, *p* = 0.003) in those with dietary fiber intake 25–29 g/day and >29 g/day, respectively (both *p* for trend < 0.001). In addition, the smooth curve also showed that dietary fiber intake was nonlinearly positively correlated with CDAI (Figure 2). As daily dietary fiber intake increased, the CDAI also increased nonlinearly.

### 3.3. Association of Dietary Fiber Intake with Risks of Death in Cancer Survivors

In all the three adjusted models, we consistently observed a strong negative correlation between dietary fiber intake and the risks of all-cause mortality and tumor mortality (Table 3). After fully adjusting for relevant factors, total dietary fiber intake was shown to reduce the risks of all-cause mortality (HR: 0.98, 95% CI: 0.96–0.99, *p* = 0.011) and tumor mortality (HR: 0.97, 95% CI: 0.94–0.99, *p* = 0.003), but was not significantly related to the risk of cardiovascular mortality (HR: 1.01, 95% CI: 0.97–1.04, *p* = 0.766) (Table 3). Subsequently, the population was stratified using the recommended dietary fiber intake and divided into <25 g/day, 25–29 g/day, and >29 g/day. After fully adjusting for other confounding factors, a fiber intake of 25 to 29 g per day reduced all-cause mortality in patients with tumors (HR: 0.52, 95% CI: 0.33 to 0.83, *p* = 0.01) (Table 3). When ingesting dietary fiber >29 g per day, tumor patients had a lower tumor mortality (HR: 0.29, 95% CI: 0.11 to 0.75, *p* = 0.01), and there was no significant association with all-cause mortality or cardiovascular mortality (both *p* > 0.05) (Table 3).

### 3.4. Association of CDAI with Risks of Death in Cancer Survivors

Similar to dietary fiber intake, high CDAI levels were inversely associated with all-cause mortality and tumor mortality in tumor survivors, and were not significantly associated with cardiovascular mortality (Table 4). According to the CDAI quartile stratified into four categories (Q1 [−6.695, −2.188], Q2 (−2.188, −0.308], Q3 (−0.308, 2.029], Q4 (2.029, 78.016]), both Q3 (HR: 0.68, 95% CI: 0.48–0.98, *p* = 0.04) and Q4 (HR: 0.56, 95% CI: 0.99–0.80, *p* = 0.001) reduced the risk of all-cause mortality compared with the lowest quartile, Q1 (Table 4). Further exploration of the association between CDAI and cause-specific mortality in cancer survivors showed that only Q4 (HR: 0.34, 95% CI: 0.16–0.74, *p* = 0.001) was associated with a reduced risk of cancer mortality and each CDAI level was not significantly associated with cardiovascular mortality.

### 3.5. Subgroup Analysis

Since smoking, drinking, high blood pressure, and diabetes are highly correlated with oxidative stress levels in the body, we stratified the population by smoking, alcohol consumption, hypertension, and diabetes. In the subgroup analysis, the benefits of dietary fiber intake and high CDAI levels mainly lay in reducing the all-cause mortality of cancer survivors, rather than tumor mortality (Supplementary Tables S1 and S2). No significant interactions were detected between dietary fiber intake and CDAI levels with these stratified variables (all *p* interactions > 0.05).

## 4. Discussion

With the spread of early screening programs for cancers and advances in therapeutic modalities, the life expectancy of cancer patients has extended, and the relative and absolute numbers of cancer survivors have both continued to increase. The number of cancer survivors in the United States is expected to increase to 22.1 million by 2030 [18]. In the face of such a large population of cancer survivors, it is necessary to develop new therapeutic strategies to improve the survival state of cancer survivors. Therefore, this study focused on tumor survivors, discussed the relationship between dietary fiber and the body’s antioxidant capacity, and explored the effect of dietary fiber intake and CDAI on the risks of death in this population. Our study demonstrated a positive relationship between dietary fiber intake and CDAI, both of which were negatively associated with the risks of all-cause death and cancer death among cancer survivors.

CDAI is a comprehensive index to evaluate the body’s dietary antioxidant capacity. Our study showed that an increased dietary fiber intake had a positive impact on CDAI, meaning that the body’s antioxidant capacity was enhanced. The mechanism of dietary fiber enhancing antioxidant capacity is complex and sophisticated [19]. On the one hand, dietary fiber is an important carrier of some antioxidant active substances, mainly the polyphenolic compounds [20]. And polyphenols, due to their special chemical structure, easily provide an electron or hydrogen to neutralize free radicals, thus have a strong antioxidant capacity [21,22]. On the other hand, dietary fiber is believed to promote the body’s absorption and utilization of antioxidants. For example, some studies have shown that high-fiber diets can improve the bioavailability of vitamin C and other antioxidants, possibly owing to the fact that dietary fiber can slow down the digestion and absorption of foods, allowing antioxidants to be fully absorbed and utilized [23]. Of course, dietary fiber itself also has a certain antioxidant activity, mainly through its decomposition product butyrate [24].

Some researchers believe that dietary antioxidant intake can slow or inhibit the development of tumors by neutralizing free radicals and repairing oxidative damage to reduce the damage caused by oxidative stress [25,26]. However, some other studies have suggested that antioxidant supplements do not improve patients’ survival and may even promote tumor metastasis [27,28]. This study supports the protective effect of dietary fiber intake against all-cause death and tumor death in cancer survivors. Especially for patients with a history of smoking, alcohol consumption, hypertension, and no diabetes, consuming 25–29 g of dietary fiber per day reduces the risk of all-cause death more significantly. This finding is similar to that observed by Song et al. in a large colon cancer cohort [29]. In addition, one recent study has also shown that dietary fiber intake can enhance the response of melanoma to immunotherapy and improve the survival status of melanoma patients [9]. This evidence suggest that increased dietary fiber intake may reduce the risk of death in cancer patients.

The possible mechanisms of this are related to the following aspects. Firstly, chronic inflammation is a common feature in cancer patients and can impair the body’s anti-tumor ability. Dietary fiber, particularly soluble fiber, produces butyrate when broken down in the colon, thus reducing oxidative stress and playing an anti-inflammatory role [24]. As a short-chain fatty acid, butyrate acts as an agonist for various G-protein-coupled receptors and can exert anti-inflammatory effects by modulating signaling pathways mediated by these receptors. For example, it can inhibit histone deacetylase and thereby influences macrophage activity, leading to the downregulation of the pro-inflammatory cytokine, such as interleukin-6 (IL-6) and IL-12p40 [30]. By reducing inflammation, it helps create a favorable immune environment, enabling the body to defend against cancer more effectively. Secondly, dietary fiber plays a crucial role in activating and enhancing the function of various immune cells, particularly immune cells in the gut-associated lymphoid tissue (GALT) region. Kelly-Quagliana et al. found that supplementation of inulin/oligofructose in mice for six weeks enhanced the cytotoxicity of natural killer (NK) cells [31]. Recent studies have discovered that a high-fiber diet promotes the colonization of certain gut microbial communities, which can effectively regulate the composition of tumor-infiltrating mononuclear phagocytes (MPs) through the STING-IFN-I pathway, improving the tumor microenvironment and enhancing the efficacy of immunotherapy [32]. Simultaneously, the breakdown product of dietary fiber, butyrate, can promote the proliferation and anti-tumor immune response of CD8+ T cells by upregulating the expression of ID2 in CD8+ T cells, thus enhancing the anti-tumor effect [33,34]. Furthermore, Qing ran Li et al. also found that butyrate can directly inhibit the phosphorylation level of Pyruvate Kinase M2 (PKM2) in colon cancer cells, enhancing PKM2 activity, and ultimately inhibiting tumor cell proliferation [35].

CDAI is a scoring system used to assess the overall antioxidant content of an individual’s diet and it can also be used to represent the antioxidant capacity of the body. Previous studies have found that CDAI levels are inversely correlated with levels of common markers of oxidative stress and inflammation, such as IL-1β and TNF-α [36]. And high CDAI levels are inversely associated with death from various causes, including cardiovascular diseases and stroke [37,38]. Our results also suggest that high CDAI levels are inversely associated with a risk of all-cause death and cancer death in cancer survivors. In addition, based on the results of the subgroup analysis, we observed that cancer survivors with a history of alcohol consumption, diabetes, and hypertension benefited more from higher CDAI levels.

Currently, one of the main approaches to enhancing the body’s antioxidant capacity is the supplementation of antioxidants, which have shown beneficial effects in certain diseases. However, it is becoming increasingly evident that more is not necessarily the better. While improving the body’s antioxidant capacity is crucial for protecting cells from oxidative stress, excessive supplements of antioxidants can also lead to a range of issues. For instance, long-term supplementation of beta-carotene (a precursor of vitamin A) was associated with an increased risk of lung cancer [39]. Besides, excessive supplementations of antioxidants may cause deleterious outcomes for some pre-existing diseases. For example, high doses of vitamin A may aggravate the condition of patients with dyslipidemia [40]. Overall, for individuals with cancer, supplements of antioxidants should be administered with caution. In some cases, the judicious use of antioxidants can enhance the body’s antioxidant capacity and may serve as a valuable strategy in combating cancer.

The advantages of our study are as follows: we are the first to assess the contribution of dietary fiber intake to the body’s antioxidant capacity. Furthermore, using the nationally representative sample data of the NHANES database, our results can be extrapolated to the national population of cancer survivors, which can provide dietary recommendations with a certain reference value for cancer survivors. Moreover, previous studies may have focused on the use of a single antioxidant to assess the influence on the survival of cancer patients, whereas our study used a more comprehensive CDAI to measure the dietary antioxidant capacity of the population. Of course, our study still has certain limitations. We only investigated the dietary data of participants at baseline. During the follow-up period, the dietary status may change with patients’ status and dietary concepts, which may bring some bias. Secondly, different tumors have different degrees of malignancy and patients receive different treatments. However, we did not classify tumors due to the wide variation in the number of tumors from different systems participating in this study. In addition, although we adjusted for many commonly known confounders, there were some unknown factors that we could not adjust. Therefore, prospective randomized controlled trials are needed for further confirmation of our findings in the future.

## 5. Conclusions

In this nationally representative study, we found that dietary fiber intake can enhance the body’s antioxidant capacity, and that an increased dietary fiber intake and CDAI level can reduce the risk of all-cause mortality and tumor-cause mortality in tumor survivors, but does not appear to affect the risk of cardiovascular death. Our results may provide new evidence for dietary and nutritional strategies for cancer survivors.

## Figures and Tables

**Table 1:** Characteristics of participants classified by survival status.

Variables	Alive	Deceased	*p* Value
Sample size	*n* = 1401	*n* = 676	
Age	61.97 ± 0.47	72.15 ± 0.51	**<0.001**
Energy (kcal)	1962.67 ± 26.71	1857.48 ± 38.63	**0.020**
Protein (g)	76.77 ± 1.10	72.76 ± 1.43	**0.019**
Carbohydrate (g)	231.11 ± 3.49	227.47 ± 5.00	0.534
Total sugars (g)	100.98 ± 2.07	104.81 ± 3.77	0.385
Total fat (g)	77.44 ± 1.25	72.27 ± 1.86	**0.019**
Cholesterol (mg)	269.98 ± 6.70	266.41 ± 8.99	0.753
Dietary fiber (g)	17.67 ± 0.38	16.21 ± 0.44	**0.005**
CDAI	0.60 ± 0.15	−0.12 ± 0.17	**<0.001**
Sex			
Male	651 (44.03%)	391 (55.49%)	**0.002**
Female	750 (55.97%)	285 (44.51%)	
Race			**0.003**
Black	206 (5.14%)	94 (7.27%)	
White	958 (87.33%)	534 (88.96%)	
Mexican American	85 (1.94%)	20 (1.16%)	
Other	152 (5.58%)	28 (2.60%)	
Education level			**<0.001**
Less than High School	102 (3.27%)	105 (12.03%)	
High School	430 (24.45%)	273 (37.28%)	
Some College or AA degree	869 (72.28%)	298 (50.68%)	
BMI			0.050
18.5–25	332 (27.70%)	206 (27.24%)	
<18.5	14 (0.96%)	16 (2.86%)	
≥25	1055 (71.34%)	454 (69.90%)	
Alcohol user			**<0.001**
Never	189 (10.89%)	91 (12.18%)	
Former	305 (18.67%)	257 (33.95%)	
Mild	615 (44.01%)	249 (41.16%)	
Moderate	179 (17.05%)	45 (6.27%)	
Heavy	113 (9.38%)	34 (6.43%)	
Smoke			**0.009**
Never	666 (45.58%)	233 (34.49%)	
Now	184 (14.57%)	100 (16.79%)	
Former	551 (39.85%)	343 (48.72%)	
Hypertension			**<0.001**
No	534 (43.07%)	178 (24.08%)	
Yes	867 (56.93%)	498 (75.92%)	
Diabetes			**<0.001**
No	899 (70.14%)	388 (58.58%)	
IFG	73 (4.94%)	28 (4.44%)	
IGT	77 (5.23%)	33 (4.14%)	
DM	352 (19.69%)	227 (32.84%)	
CHD			**<0.001**
No	1302 (93.97%)	565 (82.78%)	
Yes	99 (6.03%)	111 (17.22%)	
Stroke			**<0.001**
No	1322 (96.62%)	580 (87.68%)	
Yes	79 (3.38%)	96 (12.32%)	

CDAI = composite dietary antioxidant index; AA = associate of arts; BMI = body mass index; IFG = impaired fasting glucose; IGT = impaired glucose tolerance; DM = diabetes mellitus; CHD = coronary heart disease. *p* value in bold indicates statistical significance.

**Table 2:** Association between daily dietary fiber intake and CDAI.

Variables	Model 1		Model 2		Model 3	
Dietary fiber intake (g)	β (95% CI)	*p*	β (95% CI)	*p*	β (95% CI)	*p*
	0.28 (0.17, 0.38)	**<0.001**	0.27 (0.16, 0.38)	**<0.001**	0.24 (0.08, 0.40)	**0.004**
Categories						
<25	ref	ref	ref	ref	ref	ref
25–29	2.9 (2.06, 3.75)	**<0.001**	2.75 (1.90, 3.60)	**<0.001**	1.2 (0.37, 2.02)	**0.004**
≥29	4.15 (2.53, 5.78)	**<0.001**	4.06 (2.46, 5.65)	**<0.001**	1.99 (0.75, 3.24)	**0.003**
*p* for trend		**<0.001**		**<0.001**		**<0.001**

Model 1: Adjust for age, sex, race, and education level. Model 2: Additionally adjust for smoking and alcohol use on the basis of Model 1. Model 3: Additionally adjust for carbohydrate, energy, cholesterol, protein, and total fat on the basis of Model 2. ref = reference. *p* value in bold indicates statistical significance.

**Table 3:** Association of dietary fiber intake with risk of death in cancer survivors.

Variables	Model 1		Model 2		Model 3	
All-cause mortality	HR (95% CI)	*p*	HR (95% CI)	*p*	HR (95% CI)	*p*
Dietary fiber intake	0.977 (0.965, 0.990)	**<0.001**	0.983 (0.972, 0.995)	**0.006**	0.975 (0.957, 0.994)	**0.011**
Categories (g)						
<25	ref	ref	ref	ref	ref	ref
25–29	0.666 (0.426, 1.041)	**0.009**	0.563 (0.366, 0.867)	**0.009**	0.544 (0.338, 0.876)	**0.012**
≥29	1.063 (1.050, 1.076)	0.075	0.753 (0.491, 1.154)	0.193	0.726 (0.453, 1.162)	0.182
*p* for trend		**0.022**		0.066		0.086
Cancer mortality	HR (95% CI)	*p*	HR (95% CI)	*p*	HR (95% CI)	*p*
Dietary fiber intake	0.970 (0.953, 0.988)	**0.001**	0.979 (0.963, 0.995)	**0.009**	0.965 (0.942, 0.988)	**0.003**
Categories (g)						
<25	ref	ref	ref	ref	ref	ref
25–29	0.571 (0.296, 1.100)	0.094	0.626 (0.311, 1.260)	0.189	0.501 (0.224, 1.120)	0.092
≥29	0.294 (0.113, 0.765)	**0.012**	0.324 (0.131, 0.802)	**0.015**	0.285 (0.108, 0.751)	**0.011**
*p* for trend		**0.005**		**0.007**		**0.007**
Cardiovascular disease mortality	HR (95% CI)	*p*	HR (95% CI)	*p*	HR (95% CI)	*p*
Dietary fiber intake	0.985 (0.949, 1.021)	0.403	0.999 (0.963, 1.036)	0.958	1.006 (0.969, 1.044)	0.766
Categories (g)						
<25	ref	ref	ref	ref	ref	ref
25–29	0.811 (0.243, 2.704)	0.733	0.858 (0.298, 2.475)	0.777	0.912 (0.277, 3.005)	0.880
≥29	0.932 (0.380, 2.289)	0.878	1.225 (0.515, 2.914)	0.646	1.356 (0.665, 2.762)	0.402
*p* for trend		0.799		0.753		0.502

Model 1: Adjust for age, sex, race, and education level. Model 2: Additionally adjust for smoking, alcohol intake, diabetes, hypertension, stroke, and coronary heart disease on the basis of Model 1. Model 3: Additionally adjust for BMI, carbohydrate, energy, protein, total fat, and CDAI on the basis of Model 2. HR = hazard ratio; BMI = body mass index; CDAI = composite dietary antioxidant index; ref = reference. *p* value in bold indicates statistical significance.

**Table 4:** Association of dietary fiber intake with risk of death in cancer survivors.

Variables	Model 1		Model 2		Model 3	
All-cause mortality	HR (95% CI)	*p*	HR (95% CI)	*p*	HR (95% CI)	*p*
CDAI	0.95 (0.92, 0.98)	**<0.001**	0.96 (0.93, 0.99)	**0.008**	0.94 (0.91, 0.98)	**0.004**
Classification						
Q1	ref	ref	ref	ref	ref	ref
Q2	0.78 (0.56, 1.07)	0.127	0.76 (0.56, 1.04)	0.091	0.74 (0.53, 1.03)	0.077
Q3	0.70 (0.50, 0.99)	**0.043**	0.77 (0.56, 1.05)	0.099	0.72 (0.50, 1.03)	0.071
Q4	0.60 (0.44, 0.82)	**0.001**	0.66 (0.49, 0.88)	**0.005**	0.63 (0.44, 0.89)	**0.010**
*p* for trend		**0.001**		**0.008**		**0.003**
Cancer mortality	HR (95% CI)	*p*	HR (95% CI)	*p*	HR (95% CI)	*p*
CDAI	0.93 (0.87, 1.00)	**0.040**	0.94 (0.88, 1.00)	0.050	0.90 (0.83, 0.99)	**0.020**
Classification						
Q1	ref	ref	ref	ref	ref	ref
Q2	0.59 (0.29, 1.20)	0.145	0.58 (0.29, 1.17)	0.138	0.54 (0.25, 1.18)	0.121
Q3	0.67 (0.32, 1.40)	0.286	0.71 (0.35, 1.45)	0.333	0.60 (0.26, 1.37)	0.228
Q4	0.45 (0.25, 0.82)	**0.009**	0.50 (0.29, 0.88)	**0.023**	0.41 (0.20, 0.88)	**0.021**
*p* for trend		**0.039**		**0.042**		**0.024**
Cardiovascular disease mortality	HR (95% CI)	*p*	HR (95% CI)	*p*	HR (95% CI)	*p*
CDAI	0.95 (0.89, 1.01)	0.114	0.98 (0.93, 1.04)	0.516	0.98 (0.90, 1.06)	0.567
Classification						
Q1	ref	ref	ref	ref	ref	ref
Q2	0.76 (0.44, 1.31)	0.325	0.71 (0.43, 1.16)	0.169	0.70 (0.42, 1.17)	0.169
Q3	0.73 (0.36, 1.48)	0.378	0.80 (0.40, 1.58)	0.520	0.84 (0.36, 1.93)	0.679
Q4	0.72 (0.39, 1.32)	0.287	0.88 (0.51, 1.53)	0.645	0.88 (0.46, 1.67)	0.695
*p* for trend		0.332		0.782		0.899

Model 1: Adjust for age, sex, race, education level. Model 2: Additionally adjust for smoking, alcohol intake, diabetes, hypertension, stroke, and coronary heart disease on the basis of Model 1. Model 3: Additionally adjust for BMI, carbohydrate, energy, protein, and total fat on the basis of Model 2. HR = hazard ratio; BMI = body mass index; CDAI = composite dietary antioxidant index; ref = reference. *p* value in bold indicates statistical significance.

## Data Availability

The datasets for this study can be found in the National Health and Nutrition Examination Surveys database (https://www.cdc.gov/nchs/nhanes/index.htm) (accessed on 22 March 2023).

## Supplementary Materials

The following supporting information can be downloaded at: https://www.mdpi.com/article/10.3390/nu15132968/s1, Supplementary Table S1: Weighted multivariable-adjusted hazard ratios between dietary fiber classification and all-cause mortality and tumor mortality in subgroups of tumor survivors; Supplementary Table S2: Weighted multivariable-adjusted hazard ratio between CDAI quartile and all-cause mortality and tumor mortality in subgroups of tumor survivors.
