# Supplementary material for: Association of Dietary Fiber, Composite Dietary Antioxidant Index and Risk of Death in Tumor Survivors: National Health and Nutrition Examination Survey 2001–2018
Source: Nutrients. 2023 Jun 29;15(13):2968. doi: 10.3390/nu15132968 (PMC10346686; doi:10.3390/nu15132968)
Supplement: Supplementary file 1 [file nutrients-15-02968-s001.zip › nutrients-2394600-supplementary.pdf]

Supplementary Table S1.

Weighted multivariable-adjusted hazard ratios between dietary fiber classification and all-cause mortality and tumor mortality in subgroups of tumor survivors.

**Character**	**<25**	**25-29**	**P.val**	**>=29**	**P.val**	**P.val for trend**	**P.val for interaction**
**All-cause mortality**							
Alcohol consumption							0.305
No	ref	0.84(0.19,3.82)	0.822	0.18(0.03,1.00)	0.050	0.297	
Yes	ref	0.41(0.21,0.79)	**0.009**	0.66(0.34,1.29)	0.226	**0.008**	
Smoking							0.116
No	ref	0.94(0.43,2.04)	0.874	0.59(0.24,1.43)	0.239	0.547	
Yes	ref	0.22(0.09,0.56)	**0.002**	0.66(0.26,1.63)	0.362	**0.007**	
Hypertension							0.465
No	ref	0.38(0.15,0.98)	**0.046**	0.39(0.14,1.11)	0.076	0.612	
Yes	ref	0.46(0.23,0.93)	**0.032**	0.71(0.34,1.51)	0.375	**0.027**	
Diabetes							0.751
No	ref	0.38(0.23,0.64)	**<0.001**	0.58(0.28,1.22)	0.148	**0.001**	
Yes	ref	0.65(0.17,2.54)	0.527	0.67(0.25,1.80)	0.424	0.370	
**Cancer mortality**							
Alcohol consumption							0.648
No	ref	1.07(0.12, 9.87)	0.948	0.14(0.01, 2.32)	0.164	0.500	

Yes	ref	0.48(0.21,1.07)	0.072	0.33(0.11,1.00)	0.051	**0.021**	
Smoking							0.225
No	ref	0.99(0.36,2.74)	0.979	0.23(0.05,1.06)	0.060	0.537	
Yes	ref	0.29(0.08,1.06)	0.060	0.36(0.10,1.31)	0.120	**0.026**	
Hypertension							0.772
No	ref	0.73(0.26,2.09)	0.556	0.43(0.08,2.33)	0.324	0.500	
Yes	ref	0.41(0.15,1.10)	0.076	0.27(0.09,0.83)	**0.023**	**0.009**	
Diabetes							0.774
No	ref	0.60(0.28,1.30)	0.193	0.29(0.08,1.09)	0.067	**0.06**	
Yes	ref	0.24(0.03,2.05)	0.186	0.48(0.11,2.15)	0.334	0.218	

Data are hazard ratio (95% CI)

Models are adjusted for age, sex, ethnicity, education, BMI, except the subgroup variable itself.

Abbreviations:

CI, confidence interval

CDAI, composite dietary antioxidant index

BMI, body mass index

Supplementary Table S2.

Weighted multivariable-adjusted hazard ratio between CDAI quartile and all-cause mortality and tumor mortality in subgroups of tumor survivors.

**Character**	**Q1**	**Q2**	**P.val**	**Q3**	**P.val**	**Q4**	**P.val**	**P.val for trend**	**P.val for interaction**
**All-cause mortality**									
Alcohol consumption									0.615
No	ref	1.07(0.42,2.69)	0.890	0.81(0.34,1.92)	0.616	0.69(0.24,2.03)	0.491	0.405	
Yes	ref	0.52(0.31,0.89)	**0.016**	0.72(0.41,1.26)	0.252	0.47(0.29,0.78)	**0.004**	**0.021**	
Smoking									0.406
No	ref	0.62(0.31,1.21)	0.159	1.07(0.58,1.95)	0.832	0.50(0.28,0.92)	**0.025**	0.107	
Yes	ref	0.56(0.29,1.06)	0.076	0.60(0.30,1.19)	0.142	0.52(0.26,1.06)	0.070	0.102	
Hypertension									0.880
No	ref	0.47(0.21,1.03)	0.059	0.77(0.35,1.70)	0.512	0.54(0.26,1.13)	0.102	0.269	
Yes	ref	0.60(0.34,1.05)	0.074	0.74(0.40,1.35)	0.318	0.49(0.28,0.87)	**0.015**	**0.035**	
Diabetes									0.374
No	ref	0.65(0.34,1.24)	0.187	0.95(0.56,1.63)	0.858	0.56(0.34,0.92)	**0.022**	0.071	
Yes	ref	0.42(0.19,0.95)	**0.037**	0.40(0.16,1.01)	0.054	0.42(0.21,0.82)	**0.012**	**0.028**	
**Cancer mortality**									
Alcohol consumption									0.324
No	ref	1.15(0.25, 5.30)	0.855	1.18(0.25,5.56)	0.832	1.49(0.30, 7.44)	0.618	0.623	

Yes	ref	0.45(0.19,1.07)	0.069	0.69(0.29,1.64)	0.399	0.38(0.20,0.72)	**0.003**	**0.034**	
Smoking									0.818
No	ref	0.52(0.16,1.68)	0.273	0.97(0.33,2.88)	0.959	0.51(0.18,1.48)	0.213	0.384	
Yes	ref	0.51(0.17,1.55)	0.229	0.64(0.23,1.80)	0.394	0.41(0.17,0.98)	**0.045**	0.116	
Hypertension									0.555
No	ref	0.36(0.10,1.24)	0.103	0.83(0.27,2.55)	0.744	0.57(0.19,1.66)	0.297	0.604	
Yes	ref	0.56(0.19,1.65)	0.292	0.69(0.25,1.93)	0.481	0.38(0.17,0.88)	**0.024**	0.064	
Diabetes									0.057
No	ref	0.64(0.27,1.51)	0.304	1.38(0.63,3.06)	0.419	0.68(0.35,1.31)	0.245	0.705	
Yes	ref	0.31(0.08, 1.20)	0.089	0.13(0.03, 0.60)	**0.010**	0.15(0.07, 0.33)	**<0.0001**	**<0.001**	

Data are hazard ratio (95% CI)

Models are adjusted for age, sex, ethnicity, education, BMI, except the subgroup variable itself.

Abbreviations:

CI, confidence interval

CDAI, composite dietary antioxidant index

BMI, body mass index
